# Perspectives and practices of health workers around diagnosis of paediatric tuberculosis in hospitals in a resource-poor setting – modern diagnostics meet age-old challenges

**DOI:** 10.1186/s12913-020-05588-6

**Published:** 2020-08-01

**Authors:** Jacquie Narotso Oliwa, Sabina Adhiambo Odero, Jacinta Nzinga, Michaël Boele van Hensbroek, Caroline Jones, Mike English, Anja van’t Hoog

**Affiliations:** 1 KEMRI-Wellcome Trust Research Programme, Nairobi, Kenya; 2 Department of Paediatrics and Child Health, University of Nairobi, Nairobi, Kenya; 3 Department of Global Health, The Academic Medical Centre, University of Amsterdam, Amsterdam, The Netherlands; 4 Amsterdam Institute for Global Health and Development, Amsterdam, The Netherlands; 5 Nuffield Department of Medicine, Centre for Tropical Medicine and Global Health, Oxford University, Oxford, UK

**Keywords:** Perspectives, Health workers, Influencers, Case detection, Diagnostics, TB, Children, Kenya

## Abstract

**Background:**

Detection of tuberculosis (TB) in children in Kenya is sub-optimal. Xpert MTB/RIF® assay (Xpert®) has the potential to improve speed of TB diagnosis due to its sensitivity and fast turnaround for results. Significant effort and resources have been put into making the machines widely available in Kenya, but use remains low, especially in children. We set out to explore the reasons for the under-detection of TB and underuse of Xpert® in children, identifying challenges that may be relevant to other newer diagnostics in similar settings.

**Methods:**

This was an exploratory qualitative study with an embedded case study approach. Data collection involved semi-structured interviews; small-group discussions; key informant interviews; observations of TB trainings, sensitisation meetings, policy meetings, hospital practices; desk review of guidelines, job aides and policy documents. The *Capability*, *Opportunity* and *Motivation* (COM-B) framework was used to interpret emerging themes.

**Results:**

At individual level, knowledge, skill, competence and experience, as well as beliefs and fears impacted on *capability* (physical & psychological) as well as *motivation* (reflective) to diagnose TB in children and use diagnostic tests. Hospital level influencers included hospital norms, processes, patient flows and resources which affected how individual health workers attempted to diagnose TB in children by impacting on their *capability* (physical & psychological)*, motivation* (reflective & automatic) and *opportunity* (physical & social). At the wider system level, community practices and beliefs, and implementation of TB programme directives impacted some of the decisions that health workers made through *capability* (psychological)*, motivation* (reflective & automatic) and *opportunity* (physical).

**Conclusion:**

We used comprehensive approaches to identify influencers of TB case detection and use of TB diagnostic tests in children in Kenya. These results are being used to design a contextually-appropriate intervention to improve TB diagnosis, which may be relevant to similar low-resource, high TB burden countries and can be feasibly implemented by the National TB programme.

## Background

The World Health Organisation (WHO) recommends Xpert MTB/RIF® assay (Xpert®) as the first-line test for bacteriological confirmation of tuberculosis (TB) in children. It provides results fast (ideally within 2-4 h) compared to the 4-6 weeks of traditional bacteriological culture [1]. Despite the potential of Xpert® to improve case detection of TB, several studies, including those undertaken in Kenya, have shown that adoption and utilisation has been low [2–5]. Kenya introduced Xpert® in 2011 and now has over 156 machines, with more coming, including the newer improved Ultra® [6, 7]. Although most Kenyan county hospitals now have the machines, there is a wide policy-practice gap. Many TB patients (especially children) do not seem to benefit-over 80% have a clinical diagnosis alone and only around 1% have documented use of Xpert® [4, 5]. Given its potential to improve diagnosis due to same day results, and the effort and resources put into making it available, it is important to try to understand the reasons for underuse of Xpert®, and to identify challenges relevant to the use of it and other newer diagnostics.

Alongside the underuse of Xpert® for the diagnosis of TB in Kenyan county referral hospitals, studies have found a general problem with the diagnosis of TB among children-very few children with suggestive symptoms get a proper history and examination as per the TB guidelines [5, 8]. Documented constraints to diagnosing TB in children include the fact that TB mimics many other childhood illnesses; difficulty in obtaining suitable specimens; and low sensitivity of available tests leading to low adoption and poor utilisation [4, 5, 9–12]. A qualitative study from Peru (a high TB burden, low-resource setting) also identified constraints to diagnosing TB in children related to ignorance and stigma; limited access to diagnostic tests; inadequately trained health centre staff; and provider shortages [13]. While several studies have highlighted the challenges of implementing Xpert® among adults, data addressing constraints to the use of this diagnostic among children are sparse [3, 14–16].

New diagnostics like Xpert® are introduced into “old systems” which have established patterns of practice/behaviour that influence technology adoption [17]. Understanding these “old systems”-the context into which the new equipment is introduced, is essential to identifying practices and behaviours that are likely to enhance or constrain uptake and use. This study built on quantitative descriptions of TB detection and management in Kenyan public hospitals [4, 5] and aimed to understand how context influences/shapes TB case detection and use of TB diagnostic tests including Xpert® in children within these hospitals. It was undertaken as part of a larger body of work describing the epidemiology, adherence to clinical guidelines and use of diagnostics for childhood TB in Kenya to develop theory-driven contextually-appropriate interventions to address the gaps in detection of TB in children by targeting potentially modifiable influencers (Additional file 1).

## Methods

### Study setting

In Kenya, healthcare is organised in the following levels: i) Level 1 (Community health services) - responsible for health promotion and early identification of cases to be managed at higher levels; ii) Level 2 (Primary care services) - dispensaries and health centres that carry out preventive and basic curative services; iii) Level 3 (County referral services) - hospitals that provide more comprehensive secondary level care: where TB ideally should be confirmed as they have Xpert® machines; iv) Level 4 (National referral services) - hospitals that provide highly specialised services at tertiary referral level [18]. The National TB Programme (NTP) is responsible for TB health policy and financing; quality assurance and standards; TB health information, communication and technology amongst other administrative roles and these include paediatric TB. The NTP supports all levels of care, from the community up to tertiary level. The NTP is also responsible for training and capacity building on TB for health workers and the child TB training they offer is mainly didactic and runs for 3 days covering 10 modules covering epidemiology, diagnosis, treatment, HIV co-infection, drug resistant-TB, nutrition, monitoring and evaluation. This study focused on county hospitals and the National TB programme.

### Study design

This was an exploratory qualitative study using an embedded case study approach [19], where the broader ‘case’ of TB policy implementation (investigated at national level), and individual case studies (in-depth studies at the hospitals) are embedded within the study. It was designed to provide an understanding of the reasons for the low case detection of TB in children and minimal use of available diagnostic tests including Xpert® in Kenya [4, 5].

### Study process, sampling and data collection

The study ran from November 2017 to August 2018 in two phases.

#### Phase 1

Data were collected during regional paediatric TB sensitisation meetings; national level paediatric training workshops; meetings involving 13 hospitals that are part of a Clinical Information Network (CIN); and interviews with purposively selected staff from the 13 CIN hospitals as well as national level stakeholders. The CIN is a partnership established in 2013 between the Kenya Medical Research Institute (KEMRI), Ministry of Health (MoH) Kenya and the Kenya Paediatric Association (KPA) [20, 21]. The network collects standardized routine data on paediatric admissions from participating hospitals with the aim of promoting adoption of evidence-based interventions and improving quality of care, with documented successes over time [22–24].

Data collection involved face-to-face semi-structured interviews, small group discussions, face-to-face key informant interviews and observations of child TB trainings, sensitisation meetings and policy meetings, as well as desk review of relevant guidelines, job aides and policy documents.

There were two regional paediatric TB sensitisation meetings during the study period (day long, with 20–30 health workers from various counties sharing guideline updates and discussing diagnostic challenges) and two paediatric TB trainings (3 days long, 20 participants each). The lead investigator (JNO) was both a participant and observer in these sessions and both she and SAO recorded notes from observations of the discussions during and after the sessions.

Two regularly scheduled CIN meetings to discuss quality of care in the network hospitals were also held during the study period (each was half a day with 20 participants) and JNO and SAO attended these meetings and recorded notes from the discussions. Data on reported TB diagnostic practices and adherence to guidelines were obtained from interviews with health workers from the 13 CIN hospitals and national level TB stakeholders. Interviewees were purposively selected from among mid-level and senior managers from the CIN hospitals and officers from the National TB programme. They included paediatricians, medical officers, nurse managers and public health officers, selected due to their roles in management to explore perspectives of heads of wards/mid-level managers in the care of children with tuberculosis in Kenyan hospitals.

#### Phase 2

Based on emerging quantitative findings from the broader TB study [4, 5], and to further explore issues identified in Phase 1, an in-depth study of health care provider perceptions and practices was undertaken in two purposively selected busy CIN hospitals (paediatric admissions > 1000/year). Both hospitals were in counties with a high burden of TB but one hospital had low numbers while the other had high numbers of TB cases identified [7], and were selected to explore common and unique issues influencing diagnosis of TB in children. Data collection during phase 2 involved interviews, group discussions and observations of hospital practices. Different cadres of front line staff working in the two study hospitals were purposefully selected to elicit diverse perspectives from the different groups including: medical officers; clinical officers; nursing officers; medical officer interns; clinical officer interns; nursing officer interns and laboratory technologists.

Data collection involved JNO and SAO spending 2 weeks in each hospital attending and observing ward rounds, visiting outpatient departments, making observations to orientate to the context, as well as having informal discussions and semi-structured interviews with staff to describe what typically happens to children presumed to have TB in their hospitals.

Each formal interview took on average between 30 and 45 min. The topic guide was flexible to allow deeper exploration of issues as they arose within an interview and in subsequent interviews (see Additional file 2). The questions sought explanations/different perspectives from the different settings, freely exploring various health workers’ perceptions and experiences in diagnosing TB in children and use of TB diagnostic tests including Xpert®. Data collection proceeded until no new concepts emerged (theoretical saturation). Both JNO and SAO conducted the interviews and discussions in English, singly and sometimes in pairs. Sessions were audio-recorded using encrypted digital recorders after obtaining informed consent. Interviews and discussions were transcribed verbatim, and transcriptions reviewed for accuracy by JNO, SAO and JN. Fieldwork notes, reflections and summaries were written by both JNO and SAO to capture insights and used to understand the context, and to triangulate findings from the interviews, observed meetings, informal conversations and observations at the hospitals.

### Analysis process

**Fig. 1:**
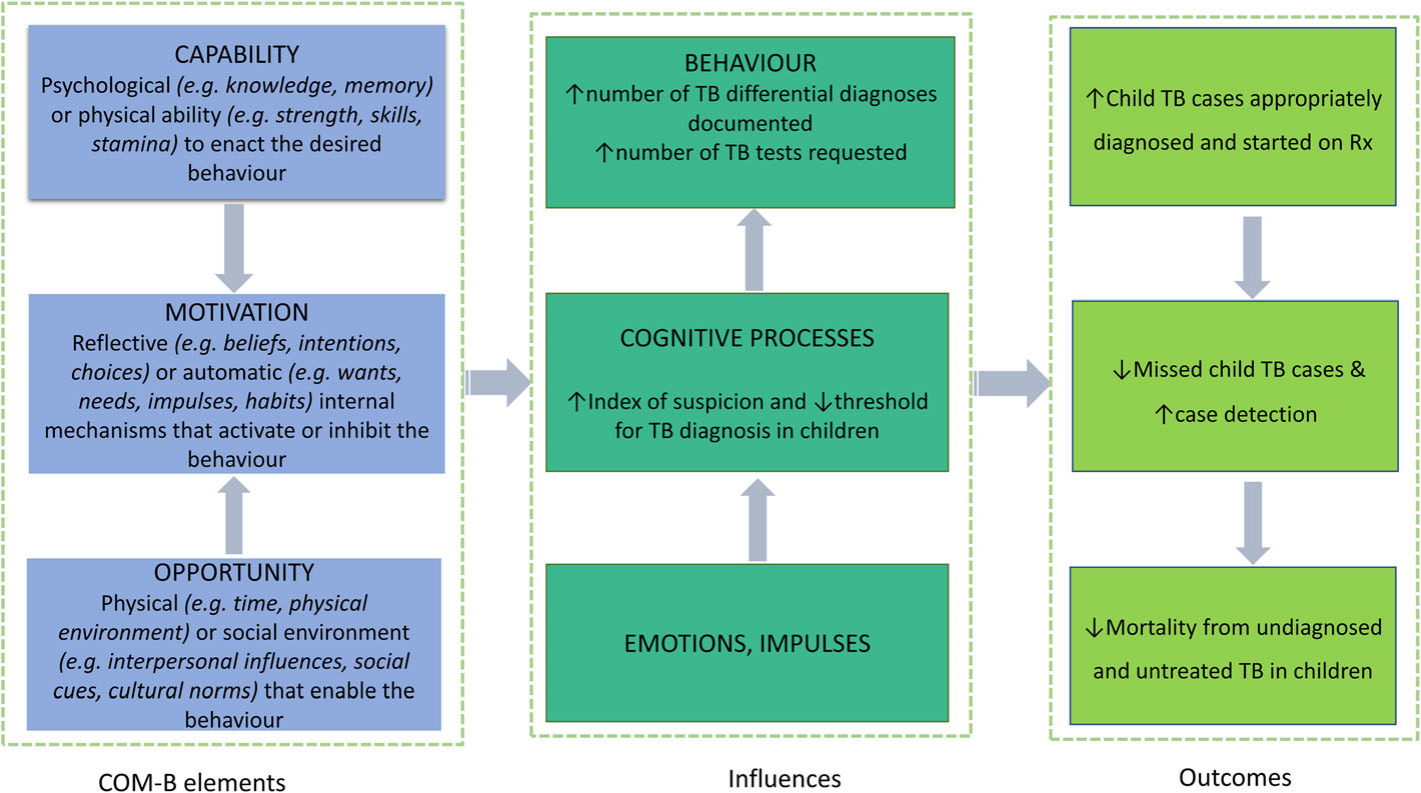
COM-B model elements, influences and outcomes

JNO reviewed all the interview and field notes’ transcripts to gain a sense of the data, then used an iterative, framework analysis approach [25] to code. Descriptive open codes were used initially and these were subsequently grouped into broad emerging themes. Charting was used to organise the emerging themes into analytic categories guided by a theoretical framework (see Fig. 1 and Additional file 3). SAO and JN also independently coded 30% of the documents to ensure consistency as part of primary coding. Using an iterative process, the investigators met over the study period to review the coding framework, resolve any discrepancies and to reach consensus. JNO identified sets of illustrative quotes for each analytic category from the coded segments and discussed with co-investigators to select the most salient. The whole team was also involved in discussions moving from descriptive to analytic coding and they approved the final groupings. The data were organised using QDA Miner® vs 5 to help with the coding and grouping and Microsoft Excel® for charting.

### Theoretical framework

We used the *Capability, Opportunity, Motivation*- Behavioural model (COM-B) framework [26] to help guide in the interpretation of the data. COM-B posits that three essential conditions interact to generate a desired behaviour/action (Fig. 1). *Capability* represents the aptitude to engage and has *physical* (e.g., strength, skills, stamina) and *psychological* (e.g., knowledge, memory) domains. *Opportunity* represents environmental factors that affect one’s capacity to perform and has *physical* (e.g., time, physical environment) and *social* (e.g., interpersonal influences, social cues, cultural norms) domains. *Motivation* represents internal factors that allow one to employ *capability* and *opportunity* to perform, and has *automatic* (e.g., wants, needs, impulses, habits) and *reflective* (e.g., beliefs, intentions, choices) domains. COM-B has been widely used in various contexts, both as a lens to analyse implementation barriers and facilitators and to help design interventions [27–47]. It was used in this work to interpret the factors that influence diagnostic practices, in a way that will enable design of interventions that might change behaviours to increase TB case detection in children and to help policy makers and health workers (key target audiences) understand the issues being explored.

### Assurance of analytic rigour

There was active engagement with participants before and over the study period. Informal discussions during clinics and ward rounds and observations of practices during the two-week hospital visits helped to triangulate findings from the formal interviews, offering explanations for observed convergence and divergence of opinions. The quantitative data from earlier work [4, 5] helped identify divergent hospitals as cases and highlighted problem areas in the cascade of paediatric TB care that were used to guide interviews. Theory was used for interpretive analysis of the data. Common and contrasting issues from different cadres of health workers were identified, noting any deviant cases. Notes were kept of observations and reflections. The Principal Investigator (JNO) being a paediatrician, member of the Ministry of Health paediatric TB technical working group and a paediatric TB trainer had an insider perspective of child TB activities in the country and found that her position made it easier to enter spaces and to get people to open up about their experiences; while SAO was a research assistant with a background in environmental health and she had a more open mind going in to interview and asking more questions for her own understanding that made the data richer. Regular peer debriefing with senior co-investigators and social science experts in the research team at various stages of data collection and analysis helped ensure reflexivity. Preliminary results were fed back to some of the participants and to child TB stakeholders during network meetings and technical working group meetings for member checking. The Consolidated criteria for reporting qualitative studies (COREQ) checklist [48] was used for further quality assurance.

## Results

Between November 2017 and August 2018, we conducted 29 semi-structured interviews with front line health workers and mid-level managers. We also held three small group discussions and five key informant interviews with policy makers and senior health service administrative staff (Additional file 4 for summary of participants). Observation notes were obtained from the 2 day-long paediatric TB sensitisation meetings, two CIN meetings, two paediatric TB trainings and 2-week visits to the two hospitals of interest. In our interviews and discussions with the various cadres of health-workers about their experiences diagnosing TB in children and using diagnostics including Xpert®, participants described at length the challenges they faced but also provided some suggestions on what could potentially be done to improve the situation.

Context: Our observations helped provide an understanding of the context of typical Kenyan county hospitals. In brief, the hospitals selected were both very busy (> 1000 paediatric admissions a year) and came from counties that reported a high incidence of TB. We noted that they had similar constraints in terms of low staffing, periodic stock-out of Xpert® cartridges and reagents and bottlenecks in work flows. The main distinguishing factor in the hospital that detected more TB cases was their localized norms and culture of teaching, mentorship and teamwork. Our observations also helped describe the flow that a patient presumed to have TB would be processed in a typical county hospital (Additional file 5).

Findings were summarised into 25 themes, representing the factors that influence TB case detection in children (chart in Additional file 3). These themes were then grouped into eight broad analytic categories, illustrating how the emerging themes had potential to impact *Capability*, *Motivation* and/or *Opportunity* to diagnose TB in children, and whether the influences were at individual, hospital or community level and are further described in the subsection that follows.

### Individual level influences

#### Knowledge and skills

Knowledge/awareness of paediatric TB did not appear to be a major challenge: most health workers across the cadres were aware of the manifestations of TB in children and how to arrive at a TB diagnosis. Most were also aware of the Kenyan paediatric TB guidelines and had had some form of TB-specific training, either from medical school or on- the- job training:

*“ … So, after you’ve enquired everything, contact with the person, loss of weight, see those things actually lead you to TB … since they have a cough and all that you’ll do a chest x-ray. A chest x-ray might actually show … you might get a miliary picture or something like that. So, after that you can do the skin test but here we don’t do it, but we do sputum for Xpert. So, we do the sputum and if it comes back positive we treat the baby for tuberculosis…”* Clinical Officer Intern_SSI_21

Many participants, both junior and senior however reported difficulties in actual specimen collection, as illustrated in the following comment:

*“…the biggest problem is specimen collection. It’s invasive, whether you are doing gastric, bronchoalveolar, because most of them … those are the things … . it’s not very easy…”* Paediatrician_SSI_10

According to most participants and what we observed, training provided by the National TB programme and other partners was mainly didactic with little opportunity to gain competence in specimen collection. Some participants therefore suggested a review of the content of paediatric TB training and how it is delivered, and this feedback was given to the National TB programme representatives.

#### Experience, confidence and competence

Where TB was more commonly detected in children, the health workers were not only knowledgeable but seemed more alert to the possibility of the disease, possibly because they had increased confidence and greater individual experience of investigating and diagnosing TB in children. Interestingly, this pattern seemed self-reinforcing, helping sustain efforts to identify TB as a shared local norm among health workers in that hospital:

*“It all boils down to … if you provide exposure to as many cases as possible then you’ll see actually day becoming … being as clear as day and night… it comes with experience [[okay]] it comes with skills, it comes with seeing many patients … ”* Paediatrician_SSI_01

In most places however, TB was rarely a differential diagnosis until the child had been seen several times for un-resolving diseases like pneumonia:

*“And you know when I get a first contact, like it will not hit to me that this is TB initially, I will treat first then from there the second time she comes … that is when I will think like, ooh this kid has been seen in the clinics outside, has been treated probably twice or thrice with antibiotics, I have also treated with antibiotics, but this is the fourth time the baby is back with a cough and a fever.”* Clinical Officer_SSI_23

Where health workers experienced marked improvement in children in whom they decided to start anti-TB medication, this affirmed their decision, making them more likely to consider TB as a diagnosis in the future (positive feedback):

*“Positive experiences … getting a child who’s doing very bad, send to nutrition, child not improving … . the moment you initiate anti-TBs, the third week, the fourth week the child is good, putting on weight. You see that child and you feel so encouraged and you’d really want to see, even if it’s a hundred and one you’ll still see tomorrow…”* Clinical Officer_SSI_31

Reflecting on their experience in using Xpert® in children, many health workers from the meetings and the various hospitals reported to have never actually never seen a positive test result:

*“I have never gotten a positive GeneXpert … All of them. In my many years by the way, I have never gotten a positive GeneXpert in our work place…”* Paediatrician_SSI_09

Consequently, the clinicians both junior and senior, had little faith in the diagnostic test, leading them to rely on their clinical acumen and treat presumptively:

*“ … especially even that GeneXpert I’ve told you it usually doesn’t help much but we have also had, you know, those x-rays sometimes you are not sure…But when you are in that dilemma you do … you give treatment and see what happens…”* Paediatrician_SSI_07

*“ … Never, never … I don’t know if it’s our samples that don’t have enough bacteria, I don’t know what the problem is, but it’s never positive. Even in someone who you are so sure this can’t be anything else … this is TB. Lakini [but] Xpert is showing you negative. We usually just continue treating as a presumptive … ”* Medical officer_SSI_24

#### Fears and beliefs

Alongside perceived competencies, some individuals also held certain beliefs or fears that influenced their practices, including the fear of acquiring TB:

*“…one of the things people [health workers] fear is getting sick. Because, you know, once you see how the TB patients struggle, finishing the 6-month medication, if you fail you have to roll over and get in your drug resistant medication. It’s crazy … ”* Public Health Officer_KII_02

In addition, for some, there was a reluctance to diagnose and treat TB in children linked to a fear of the side effects of the drugs:

*“ … We didn’t treat because we were afraid that…the liver was an issue. I think we learnt that we should treat regardless … ”* Medical Officer_SSI_32

Relatedly, the reluctance to diagnose was often linked to underlying beliefs that children do not usually get TB as shown below;

*“To be honest, I think I have been a bit reluctant. I’ve not been that vigilant to identify this child [ren] with TB, which I’ll start from now … We are so reluctant on our part. Or maybe we may overlook these children; maybe we may think … we may not suspect a child may be having TB … ”* Nursing Officer_SSI_30

However, as described above, fears were allayed as health workers observed children improving with treatment:

*“I don’t think I have that fear anymore in terms of saying yes, start this child on anti-TB. I think our confidence levels with time and having observed children, you know there are some you see, you start on anti-TB then the improvement within a month or two is like magical…”* Paediatricians_SGD_11

These examples show that at individual level, experiences affect one’s knowledge, skill and competence and can increase or decrease one’s perceived *capability* (psychological) to make a child TB diagnosis. This is often reinforced by positive experiences of improvement where treatment had been initiated and in other cases discouraged by negative test results. Positive experiences therefore contribute to the health worker’s *motivation* (reflective) to keep trying to diagnose TB in children, especially if they can see or receive news of the clinical results of their practice. This in turn affects their *opportunity* (social), because no culture of Xpert® use is established and so they fail to gain competence. Strongly held fears and beliefs about TB possibly affected *motivation* (reflective & automatic) negatively.

### Hospital influences

#### Hospital norms

In the hospital that reported higher numbers of child TB cases, established localized norms guided work practices. Senior clinical leads offered teaching and mentoring, fostered multi-professional teamwork, with every member having shared responsibility for ensuring patient well-being; and National TB guidelines used as standard practice. These local working practices enhanced individual capabilities as they created a conducive environment where good practices were taught and encouraged:

*“When you get to the ward you are trained and now you are the one who will be getting it … They definitely teach us… Clinical officer [X] is very helpful … he’s the one who taught us how to collect the sputum after Dr [M] had taught us … he also repeated the whole thing as in physically…”* Clinical Officer Intern_SSI_21

A key feature of this conducive environment was facilitative teamworking where team members relied on each other, for example, in making a diagnosis:

“ *… then since us we are interns we have people who are more experienced than us … the clinical officers … and the MO* [Medical Officer] *who is in paediatrics and also Dr. K so you just talk to … your immediate-most senior like an MOI* [Medical Officer Intern] *if he’s unable … we talk to our MO and then maybe them they can do it* [specimen collection] *…”* Clinical Officer Intern_SSI_21

Of note in several hospitals, leadership and mentorship was missing as some senior clinicians were not at-ease doing specimen collection procedures themselves. This lack of competence by seniors consequently lead to challenges in diagnosing TB in children:

*Interviewer: “Have you ever participated in the sample collection process?”*

*Respondent: “No. I am used to giving instructions and go. Maybe now I should participate to see how it is being done. Because I am now suspecting, could it be the sample collection which is causing the issue?”* Paediatrician_SSI_07

#### Organisational processes and resource management

In the hospitals we visited, we noted there was poor patient flow, no designated procedure on when or where investigations should be done for children seen in the outpatient department of both hospitals (patient flow process map Additional file 5). Consequently, as reported by some of the participants, this led to a lack of continuity of care:

*“…So, I was feeling the challenge that is there in making the diagnosis of TB is that when the child leaves here, you don’t know when … if the child is going to the next … will get to the next place, and if they are going to have a Mantoux [TB skin test] done, is the report going to come back to you? You know if it doesn’t come back to you directly, you’ll find … the child might get lost somewhere along the way … because if you are not the same person seeing that same patient again, you don’t know what the decision of the next person will be. And you’ve sent them for a Mantoux, the interpretation, who will interpret it and are they going to use the same thought that you had?”* Clinical Officer_SSI_24

The lack of proper post-discharge follow-up was common in most of the other hospitals:

*“Now there is a gap in this child at out-patient at this level, once treatment is initiated in the ward the child is discharged. Linking them to a TB clinic sometimes becomes an issue, so they may fall by the way side, they may not end up in the TB clinic, or they may interrupt TB treatment because of that…”* Public Health Officer_KII_01

Where key resources were available (equipment; reagents; skilled manpower; guidelines/job aides), clinicians could more comfortably make a TB diagnosis (*psychological capability and motivation)*, like in the following example:

*Respondent 2: “ … yes, yes, yes. There are some charts even in the nursing station I think you’ve seen one. There is a chart on the wall. Yeah, but basically as she has said, these are things we do almost every day so most of them actually stick…”*

Respondent 2*: “ … any time you think you have forgotten something. You know there is the paediatrics bible, that is the paediatrics protocol … ”* Small group discussion with interns

However, where there were resource shortages, health workers struggled:

*“Against us again is the X-rays, because X-rays are a mainstay of diagnosis for TB in children. Unfortunately, they have not been readily available all over the country. They are available in very few sites and in those sites, there is a cost implication to the children which sort of acts as a deterrent or a limitation to the same…”* Public Health Officer_KII_01

For the diagnostic tests, commonly reported issue was frequent stock-out of Xpert® cartridges and reagents (nation-wide) which in turn led to delays in making a diagnosis and reinforced a reluctance in ordering the tests in future. This shows how age-old system issues like stock-outs potentially affect adoption of new diagnostics:

*“ … Most times no coz sometimes we have stock outs of Xpert … when there are stock outs you might want to send the patient to another place, where … maybe a private facility where they have to pay for it out of pocket … ”* Medical Officer_SSI_14

The influences of hospital capacities in diagnosing TB therefore span hospital norms including multi-professional teamwork, leadership and mentorship; as well as processes and resource management. The hospital environment thus affected both group and individual work practices around diagnosing TB in children by influencing *opportunity* (physical & social) which in turn affects psychological *capability* as well as *motivation* (reflective) to keep at it.

### Community influences and implementation of policies and directives

Beyond individual and hospital levels, we identified themes spanning two aspects of the broader health system: the policy level and characteristics of the population seeking care.

#### Community beliefs and practices

Stigma, health-seeking behaviour and community awareness of TB manifestations in children made some health workers reluctant to test and treat for TB as illustrated in this example:

*“…in a few instances you tell the parent the child has TB and they get very mad. They don’t want to believe it, ‘You can’t say my child has TB, kwetu hakuna TB [there is no TB where come from]’ …in fact there are some who even refuse treatment arguing that their place people don’t get TB, especially the rich people … ”* Paediatrician_SSI_07

Of note, TB is stigmatised in this setting due its presumed association with HIV, which increased reluctance by health workers to give a TB diagnosis, as health workers feared it may lead to emotional burden for their patients as seen in this illustration:

*“ … And then there is that thing people thinking TB is equal to HIV, so when now someone has been told that they have TB now everyone thinks that they are HIV positive, so there is that even being shunned by the family. I have a mother right now who was actually chased away by her extended family because of the TB diagnosis…”* Paediatrician_SSI_03

#### Implementation processes by the national TB programme

At policy level, we found that that some of the National TB programme implementation decisions affected health workers’ capacity to use TB diagnostic tools. For instance, when Xpert® was being introduced in Kenya, the selection of participants to take part in trainings inadvertently left out key actors like clinicians, resulting in low demand for use of the diagnostic reported here:

*“ … We realized that when we rolled out Xpert, we focused a lot of our training on the lab personnel, thereby leaving out the drivers of the service use. So, the clinicians initially were not part of the target population for training and so what we have realized as a programme is that therefore the demand for the service is skewed and is not actually being availed to the people who need the service … ”* Public Health Officer_KII_01

Policy -related directives from the National TB programme that encourage data use for audit purposes could subsequently motivate quality improvement initiatives in hospitals which lead to increased number of children diagnosed with TB:

*“ … But in terms of feedback … we do data quality audits and they are done together with the health care workers so it is a participatory sort of quality audit. And the feedback* [about performance] *is given on the spot … ”* Public Health Officer_KII_02

We therefore found that health worker practices are influenced by what was happening in the wider communities and from policy implementation processes led by the National TB programme which affect the *opportunity* (physical) and *motivation* to diagnose TB in children.

**Fig. 2:**
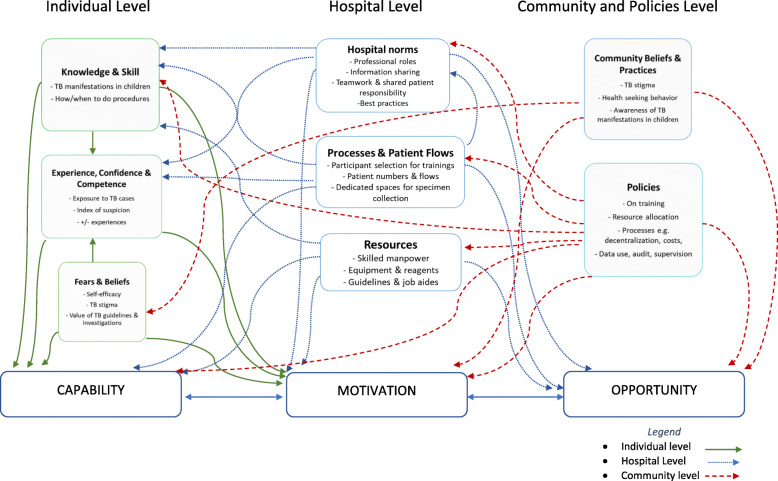
Emerging themes showing connections with each other and COM-B elements

In summary, we have described influencers of diagnosing TB in children at different levels: (individual, hospital and the wider community and policy level) and shown how these factors interact to influence the behaviour of health workers through impacting *capability, opportunity* and *motivation* (illustrated in Fig. 2 and chart in Additional file 3). At individual level, knowledge, skill, competence and experience, as well as beliefs and fears impacted on *capability* (physical & psychological) to diagnose TB in children and use diagnostic tests, and eventually their *motivation* (reflective then automatic) to keep doing it will lead to sustained practice. Most of the issues of processes and resources at hospital level we thought had potential to impact *capability* (physical & psychological) and *opportunity* (physical & social), because of breaks in the care, and this in turn could influence *motivation* (reflective and eventually automatic) through impaired decision making. Community beliefs and practices as well as policies, we thought influenced *capability* (psychological), *motivation* (reflective & automatic) and *opportunity* (physical*)* and because of these, the health workers seemed hesitant/reluctant to make a TB diagnosis in children.

## Discussion

We set out to document experiences of health workers in Kenyan county hospitals, describing the context and influencers of TB case detection and use of TB diagnostic tests in children. This was to explore reasons for gaps noted in earlier studies from the same setting [4, 5, 49] and to identify potentially modifiable influencers, guided by the COM-B framework. At individual level, notable factors included knowledge, skills and competence, which were influenced by past experiences and confidence (mainly drawn from experiential knowledge and strongly held fears and beliefs about TB) and these affected *capability* (physical & psychological). Guided practice and positive experiences of success seemed likely to contribute to health workers’ *motivation* (reflective) to keep trying to diagnose TB in children, findings consistent with ideas around ‘trialability’ in the wider implementation literature and dissemination of innovations [50–52]. A notable example was consistent negative Xpert® results leading to loss of faith in the diagnostic, which probably contributed to its underuse. Difficulties in obtaining specimen and bacteriological confirmation of TB in children is a recognised age-old dilemma [53]. Although knowledge seemed adequate and most participants had had some form of paediatric TB training, this on its own was not enough to translate into changes in practice, and this supported by various other studies [54, 55]. While formal education and training of health workers is key to ensuring competence and *capability*, it is now apparent that diagnosing TB in children is mostly reliant on “embodied” or tacit knowledge, developed through observing empathetically and hands-on experience, described by various authors [56, 57]. Improving the quality of training by the National TB Programme to make it more practical and ensuring continued mentorship and sensitisation or other sustaining strategies such as supervision or group problem solving after training could potentially address some of these gaps, as described by Rowe et al. [55, 58].

At the hospital level, the influences of hospital capacities in diagnosing TB in children spanned hospital norms including multi-professional teamwork, leadership and mentorship; as well as processes and resource management. The hospital environment affected both group and individual work practices around diagnosing TB in children by influencing physical and social *opportunity* as well as *motivation* to keep at it. While resources may not be immediately modifiable, they do have great impact and should be available for anything else to work well. We saw how frequent stock-outs of Xpert® cartridges and reagents were a hindrance to developing a culture of its use. Lack of availability of resources and staffing issues are some of the age-old challenges in lower-income settings like ours, and these hinder adoption of new policies and health interventions [59]. Patient flows and processes are however potentially modifiable. We observed lengthy procedures and bottlenecks that ended up frustrating both health workers and patients. Poor patient flow has been recognised elsewhere as an impediment to quality of care given to patients [60] and simple care redesign strategies can improve patient flows using existing capacity efficiently, leading to improved physical opportunities to diagnose TB in children. We also noted in the facility with high TB case detection, the existence of positive hospital cultures and norms like teamwork, mentorship and shared responsibility for patient care provided social *opportunity*, an environment conducive to routine diagnosis of TB in children including improved processes. Schein describes organizational culture as a pattern of basic assumptions held by a group that has worked well enough to be considered valid and therefore is taught to new members as the correct way to perceive, think, and feel [61]. The cumulative way in which health-workers experience their jobs and lives at the organization is therefore a key factor in quality improvement and can potentially be leveraged to improve case detection of TB in children: mid-level managers are key [62–65].

At community level, a notable influencer was stigma, which seemed to reduce willingness to diagnose TB in children. Stigma arose from local communities-some caregivers believed that TB was a disease of poor people. Health workers themselves sometimes perpetuated TB stigma-some did not look for TB in children and seemed unwilling to diagnose it due to their own fears and beliefs. Various studies reveal the main reason for TB stigma is fear of infection (although in our case, it seemed to be mainly due to the association of TB with HIV), and that TB stigma increased diagnostic delays and treatment noncompliance [66, 67]. Addressing stigma is fundamental to delivering quality healthcare in general and needs to be factored in efforts to improve TB case detection. It is an example of how the “outer setting” i.e. the social context as described by Damschroder, influences what happens at hospital and individual level [52]. Strategies to address stigma and patient beliefs need to be multi-level (from patient-level, to the community, to policy level, to the institutions and to individual health workers) to be effective and may include structural/policy changes, patient empowerment, education, and counselling amongst others [68, 69]. Policies are also part of the outer setting. We found that some of the National TB programme implementation decisions affected health workers’ capacity to diagnose TB in children using diagnostics. We saw for instance how training directives (i.e. who gets selected for trainings) impacted hospital practices and ultimately on the individual health worker’s knowledge, skill and competence in diagnosing TB in children by providing *opportunity* and therefore impacting their *capability* and *motivation* to keep at it. Human resources for health literature suggest that even in settings like Kenya where it may be challenging to increase staff numbers, smart policies like those aimed at strengthening retention, education, training, job-protection for staff can still achieve good health outcomes [70].

Our study had several strengths. We employed various strategies to ensure rigour, including purposive selection of cases to allow comparison and a wide range of perspectives; triangulation of findings from interviews, discussions and observations; clear records of all processes; member checking; and debriefing and support from colleagues. The research was embedded in theory that helped get a better understanding of the problem of TB case detection in children as a part of a larger mixed methods study, which will help guide development of contextually appropriate interventions. This work helps extend the body of literature in which the COM-B models has been used to understand health systems and to better explain the complex problem of diagnosing TB in children.

We had some limitations. As this was baseline formative work, to be useful it needs to progress to inform plans for testable interventions. Our data collection was cross-sectional, but we still managed to delve deep into the issues by using rich and varied data collection methods. There was a lot of disruption caused by prolonged industrial action by the health workforce during the study period [71]. This influenced who was available to interview and how much time we could spend at each site. We however leveraged on good relationships from long standing quality improvement work by our group that eased participants’ willingness to work with us despite being disgruntled.

## Conclusions

We used comprehensive approaches to identify modifiable influencers of TB case detection and use of TB diagnostic tests in children in Kenya, which is a high burden setting and few children get notified to the TB programme. At individual level, knowledge, skill, competence and experience, as well as beliefs and fears impacted on *capability* (physical & psychological) as well as *motivation* (reflective) to diagnose TB in children and use diagnostic tests. Hospital level influencers included hospital norms, processes & patient flows and resources which affected how individual health workers attempted to diagnose TB in children by impacting on their *capability* (physical & psychological)*, motivation* (reflective & automatic) and *opportunity* (physical & social). At the wider system level, community practices, beliefs, and implementation of TB programme directives impacted some of the decisions that health workers made through *capability* (psychological)*, motivation* (reflective & automatic) and *opportunity* (physical). These results are being used to design a contextually-appropriate intervention to improve TB diagnosis, which may be relevant to similar low-resource, high TB burden countries and can be feasibly implemented by the National TB programme.

## Supplementary information

**Additional file 1.** Mixed Methods Conceptual Framework.

**Additional file 2.** Interview guides.

**Additional file 3.** Influencers of case detection and use of diagnostic tests.

**Additional file 4.** Summary of interviewees.

**Additional file 5.** Patient Flow in a typical Kenyan county hospital.

## Data Availability

The datasets (interview transcripts and observation notes) generated and analysed during the current study are not publicly available due to maintaining confidentiality of the study participants but are available from the corresponding author on reasonable request.

## Abbreviations

Anti-TB: Anti-tuberculosis medication
CIN: Clinical Information Network
COM-B: *Capability, Opportunity, Motivation*- Behavioural model
COREQ: Consolidated criteria for reporting qualitative studies
DST: Drug Susceptibility Testing
IPT: Isoniazid preventive Therapy
KEMRI: Kenya Medical Research Institute
KPA: Kenya Paediatric Association
MoH: Ministry of Health
MO: Medical Officer
NTP: National Tuberculosis Programme
RTI: Respiratory Tract Infection
Rx: Treatment
S/S: Signs and Symptoms
TB: Tuberculosis
WHO: World Health Organisation
