# Supplementary material for: Perspectives and practices of health workers around diagnosis of paediatric tuberculosis in hospitals in a resource-poor setting – modern diagnostics meet age-old challenges
Source: BMC Health Serv Res. 2020 Aug 1;20:708. doi: 10.1186/s12913-020-05588-6 (PMC7395417; doi:10.1186/s12913-020-05588-6)
Supplement: Supplementary file 2 — Additional file 2. Interview guides. [file 12913_2020_5588_MOESM2_ESM.pdf]

KEMRI-WELLCOME TRUST RESEARCH PROGRAMME: HEALTH WORKERS INTERVIEW GUIDE

UNDERSTANDING AND IMPROVING CASE DETECTION AND CARE FOR CHILDREN WITH TUBERCULOSIS IN KENYA

Tuberculosis (TB) is a major global public health challenge, and diagnosis in children remains a challenge. The Kenyan health care system requires all cadres of health workers to be equipped to suspect, diagnose and treat children with TB, but their capacity to diagnose childhood TB is unknown. I will ask you some questions around care provision for children with suspected TB in your facility, and would request you answer as honestly as possible. Thank you

Gender
- ☐ Male
- ☐ Female

Educational background as a health care provider
- ☐ Nurse
- ☐ Pediatrician
- ☐ Laboratory technician
- ☐ Medical Officer
- ☐ Clinical Officer
- ☐ Intern
- ☐ TB Clinic MO/CO

Other (specify) ______________________________

Type of facility/level of care e.g County referral, sub county etc ______________________________

Which department do you work in?
- ☐ Chest clinic
- ☐ MCH
- ☐ Outpatient – IMCI
- ☐ Paediatric ward
- ☐ Other, specify……………………..

How many cases of children suspected of having TB do you see on average in a:

- Day?
- Week?
- Month?

You get a child admitted with cough, difficulty in breathing, cyanosis and severe lower chest wall indrawing referred from a lower level facility after having been on Amoxil for 3 days without improving.

Please talk me through how you would provide care for such a child

Probe: what further history would you ask for?

i. Why would you ask for the particular factors you have stated?
ii. How would you establish if this child has a history of TB contact?
iii. How has your experience been in trying to determine if a child has had a history of contact?

If there are challenges (e.g. stigma) in establishing contact history, how do you deal with them?

- duration of cough
- fever
- poor weight gain
- lethargy or reduced playfulness
- Ask for history of contact with adult/adolescent with chronic cough or TB within the last 2 years.
- Contact includes frequency and duration of contact e.g. sleeping in the same room, caregiver etc

Probe: what would you look for in his physical exam?

i. Physical examination

Probe to find out what they usually check for in terms of abnormal respiratory findings. List should include: cyanosis, chest wall indrawing, fast breathing (tachypnoea), wheeze, crackles, low oxygen saturation (SPO2), acidotic breathing

- Temperature >37.5 (fever)
- Weight (to confirm poor weight gain, weight loss) - check growth monitoring curve
- Respiratory rate (fast breathing)
- Respiratory system examination - any abnormal findings

Following these history and examination findings, what differentials would you consider for this child, and why?

- URTI
- Pneumonia
- TB
- Allergic reaction
- Foreign body inhalation
- Heart disease

You suspect this child could be having tuberculosis from the history and physical examination and you need to investigate further to confirm

Probe: What investigations or tests would you do?

i. Which tests are available in your hospitals to test for TB? Which one do you prefer using?
ii. At what point would you ask for the specific investigations along the evaluation process?
iii. What has been your experience using each of the TB investigations:
    - when does the child get the test in patient (during admission or how many days after?) vs out patient.
    - who orders the tests? Where do they document?
    - can an intern order,
    - does the test result come with a report?
    - who reads the report
    - are the interviewees themselves comfortable reading the x-rays and interpret results for this and other TB tests (based on which they say they use)?

- Xpert MTB/RIF
- Chest x-ray
- Microscopy
- Culture
- Mantoux

(Probe further for Xpert. Does their hospital have a machine? Have they ever sent specimen for children? How many times past 3 months? Any positive result past 3 months? How quickly do they get results? Hours/days/weeks? Pos/Neg experience using it?)

Probe: Which type of sample would you ask for?

i. How would determine the type of specimen to collect for testing?
ii. How frequently are they collected?

- Sputum (probe for specimen)
- Other specimen e.g. FNA, bronchoscopy and bronchoalveolar lavage (BAL), biopsy, CSF, Joint aspirate, stool, urine

How would you obtain this sample? (explore other methods apart from sputum induction)

i. Who collects the specimen (here ask also if they have champions for the sample collection procedures)?
ii. What are some of the highlights/downsides to these sample collection procedures?

- Sputum expectoration
- Gastric aspiration
- Sputum induction
- Tuberculin skin test (TST)
- Nasopharyngeal aspirate
- Gastric aspirate

What has been your experience with these sample collection procedures?

i. Which methods have you personally carried out?
ii. How long did it take you?
iii. What were the good, the bad?

Probe: how would you arrive at a final diagnosis of TB in Moses?

i. When and how do you arrive at a final diagnosis of TB??

Probe how they interpret the guidelines to make:

a) clinical TB diagnosis, what combination of signs, symptoms and tests would they use?

b) Bacteriologically confirmed TB, what tests would they do?

Show them a chart of the guidelines and probe for their experience in using these guidelines. Which areas do they find challenging and why?

ii. Who makes the final TB Diagnosis decision? Can interns/medical/clinical officers make decisions to start treatment or do they have to wait for someone more senior like a paediatrician?

iii. What treatment would you start, and when would you change and why?

- **Bacteriologically confirmed TB:** Diagnose if specimen is positive for MTB
- **Clinically diagnosed TB:** diagnose if c*hild has* ***two or more*** *of the following suggestive symptoms: Persistent cough, fever, poor weight gain, lethargy* PLUS **two or more** of the following: Positive contact, abnormal respiratory signs, abnormal CXR, positive Mantoux

What has your experience been with diagnosis of childhood TB?

i. Please tell me of the negative or positive experiences you have had
ii. What lessons have you learnt from these experiences?
iii. What would you do differently?

From this discussion, these seem like a lot to remember. What strategies do you use/have in place to help you decide if a child has TB or not? How frequently do you refer to these aides?

We looked at one-year data from your medical records. In this period, we found the following:

1. Admissions to children's wards = 3,018
2. Admissions with a respiratory tract infection/pneumonia = 1,495 (49%)
3. Children with 2 or more suggestive signs and symptoms of TB amongst the RTIs = 941 (62.9% of all RTIs)
4. Children with a working diagnosis of TB (i.e. either admission/discharge Dx or were started on anti-TB) = 33 (1.08% of all admissions)
5. Children with a missed diagnosis of TB (i.e. had 2 or more suggestive symptoms, but did not get a TB test or get a working TB Dx) = 1,356 (44.4%)
6. Amongst those with a working TB Dx, 7 (21.2%) had an Xpert test; 8 (24.2%) had a chest x-ray, and no children had documentation of culture, smear or Mantoux test being done

Why do you think there were so many potential missed diagnoses?

What can be done to improve this situation?

In your opinion, why do you think there so few investigations carried out?

What can be done to improve this situation?

KEMRI-WELLCOME TRUST RESEARCH PROGRAMME: KEY INFORMANTS INTERVIEW GUIDE

UNDERSTANDING AND IMPROVING CASE DETECTION AND CARE FOR CHILDREN WITH TUBERCULOSIS IN KENYA

Tuberculosis (TB) is a major global public health challenge, and diagnosis in children remains a challenge. The Kenyan health care system requires all cadres of health workers to be equipped to suspect, diagnose and treat children with TB, but their capacity to diagnose childhood TB is unknown. I will ask you some questions around your experiences surrounding care provision for children with suspected TB, and would request you answer as honestly as possible. Thank you

Educational background as a health care provider and what additional training have you received?	______________________________
Type of facility/level of care e.g County referral, sub county etc	______________________________

Which department do you work in and what does your role entail with regards to TB in children?

How long have you served in this capacity?

What have been some of your experiences (good and bad) around case detection of TB in children as you serve in your role?

What is your opinion on the priority given to child TB by the Ministry of Health? Why is it a priority or not? How are resources allocated for your docket? What are your thoughts on this? Explain reasons why?

What targets have been set to achieve control in TB in children in your county? How far have we gotten in meeting these targets? What has been your experience in this?

We looked at one year data from medical records. In this period, we found the following:

1. Admissions to children's wards = 3,018
2. Admissions with a respiratory tract infection/pneumonia = 1,495 (49%)
3. Children with 2 or more suggestive signs and symptoms of TB amongst the RTIs = 941 (62.9% of all RTIs)
4. Children with a working diagnosis of TB (i.e. either admission/discharge Dx or were started on anti-TB) = 33 (1.08% of all admissions)
5. Children with a missed diagnosis of TB (i.e. had 2 or more suggestive symptoms, but did not get a TB test or get a working TB Dx) = 1,356 (44.4%)
6. Amongst those with a working TB Dx, 7 (21.2%) had an Xpert test; 8 (24.2%) had a chest x-ray, and no children had documentation of culture, smear or Mantoux test being done

Why do you think there were so many potential missed diagnoses? Why do you suppose there might be clinician reluctance to suspect TB, even to order tests? (from interviews, TB Dx only comes after several Rx of pneumonia)

What can be done to improve this situation?

In your opinion, why do you think there so few investigations carried out?
Some health workers have reported that getting negative results even in cases where clinically they can make a diagnosis of TB. Any idea of the numbers of negative Xperts in children? What do you think might be causing this situation? How can the situation be improved?

What specimen collection methods have you observed people here mainly use? Who is responsible for specimen collection? Decision to start TB treatment?

What is the average Turn-around time for tests? Reasons for this? Any idea of proportions of loss to follow up? Is it an indicator you track?

Who procures reagents? How do they get feedback on stock outs? What are service contracts like for broken down Xpert machines?

How well do clinical teams work together to diagnose and start TB treatment in children? Positive and negative experiences and reasons why?

Let's talk through the process a child goes through in a typical district hospital. How they would be processed up to start of TB treatment?
Probe with an example of a process map.
What are some of the bottle necks in your opinion? Why? The hospital is a complex system with a number of different departments that are involved in the care for children with TB. How can we ensure that are these departments are able to appropriately manage TB in children?

How do you ensure that guidelines get embedded in to the institutions and in to daily practice by individual health workers? How do you in practice ensure that this get done? Any M/E? how is communication/awareness about them generated? Any feedback to and from the health care workers on the same?

For child TB training, please expound/describe the training, who is selected, where are the trainings done, by whom, how often, where, how are sites selected etc. In your opinion, what are some of the gaps?

Any form of supervision/mentorship specifically for child TB? How is it done? By whom, where, how often etc. Do health care workers ever get feedback on the cases detected, data quality, lab specimen quality? How often? By whom? In your opinion, what are some of the gaps?

Finally, in your role, what is working well, what is not working well and why? what support is in place to make sure your role is effective, do you feel supported, and who supports you? What tools/structures are in place for this support. (e.g. guidelines/frameworks)? What would you like to see happen, that would improve things e.g. staffing?
Probe further, to give specifics to see how each step will address the problem.
