# Supplementary material for: Perspectives and practices of health workers around diagnosis of paediatric tuberculosis in hospitals in a resource-poor setting – modern diagnostics meet age-old challenges
Source: BMC Health Serv Res. 2020 Aug 1;20:708. doi: 10.1186/s12913-020-05588-6 (PMC7395417; doi:10.1186/s12913-020-05588-6)
Supplement: Supplementary file 3 — Additional file 3. Influencers of case detection and use of diagnostic tests. [file 12913_2020_5588_MOESM3_ESM.docx]

Charting influencers of TB case detection and use of TB diagnostic tests in children

(N/B shaded area shows which domain of COM-B each theme occupies)

| **INFLUENCERS** | **ILLUSTRATIVE QUOTES** | *Physical and psychological capacity to engage in activities to diagnose TB in children, including the necessary knowledge and skills* | | *Factors lying outside the individual that make it possible for them to diagnose TB in children* | | *Cognitive processes including habitual processes, emotional responses as well as analytic decision-making that direct activities toward diagnosing TB in children* | |
| --- | --- | --- | --- | --- | --- | --- | --- |
|  |  | **Physical capability**  *Strength, skills, stamina* | **Psychological**  **Capability**  *Knowledge, memory* | **Physical Opportunity**  *Time, physical environment* | **Social Opportunity**  *Interpersonal influences, social cues, norms* | **Reflective Motivation**  *Beliefs, intentions, choices* | **Automatic Motivation**  *Wants, needs, impulses, habits* |
| **INDIVIDUAL-LEVEL INFLUENCERS** | | | | | | | |
| **Confidence, Competence & Experience:** | | | | | | | |
| -Frequent exposure to cases | *“I would say maybe suspected TB maybe 2 a week and at minimum 1 a week…”* Paed_SSI_06 |  |  |  |  |  |  |
| -Index of suspicion of TB in children | *“this kid…has been treated probably twice or thrice with antibiotics, I have also treated with antibiotics, but this is the fourth time the baby is back...”* CO_SSI_23 |  |  |  |  |  |  |
| -Reinforcement by positive/negative experiences | *“you start on anti-TB then the improvement within a month or two is like magical...”* Small group discussion with paediatricians |  |  |  |  |  |  |
| **Knowledge and Skill:** | | | | | | | |
| - Of TB manifestations in children | *“there’s cough, there’s fever, there’s less playfulness so I’ll think of TB...”*CO_SSI_05 |  |  |  |  |  |  |
| - Of how and when to do procedures | *“for adults it’s obvious…sputum you will be able to get…For children getting the specimen is usually the most challenging thing.”* Paed_SSI_13 |  |  |  |  |  |  |
| **Fears & Beliefs:** | | | | | | | |
| - Self-efficacy | *“gastric aspirate… I’ve learnt how to do it. I think now am confident I can do it…”* CO_SSI_31 |  |  |  |  |  |  |
| - Stigma | *“people are still afraid even those already on treatment…afraid to be stigmatized...”* MO_SSI_032 |  |  |  |  |  |  |
| - Value of guidelines & investigations | *“The guidelines, fine they are there, but sometimes…you don’t use them…Most times actually…”* MO_SSI_25 |  |  |  |  |  |  |
| **HOSPITAL/INSTITUTIONAL LEVEL INFLUENCERS** | | | | | | | |
| **Hospital norms:** | | | | | | | |
| - Professional roles & leadership | *“we train interns so they are usually… the people who do most of these investigations…the intern and the medical officer”* Paed_SSI_12 |  |  |  |  |  |  |
| - Information sharing & data for decision making | *“you might get cases whereby maybe you are not really comfortable making the diagnosis for TB, but through sharing of knowledge you might get people who’ve had the same experience and you want to share and they also want to share of how they have been able to go through the same cases...”* Paed_SSI_02 |  |  |  |  |  |  |
| - Teamwork & shared patient responsibility | *“But at least in KR I think we are doing well. We have a really good team that is very good at really detecting some cases…”* Paed_SSI_08 |  |  |  |  |  |  |
| - Routine use of guidelines & best practices | *“Once they [guidelines] are launched, they take a bit of time to get there but eventually we do…”* KII_1 |  |  |  |  |  |  |
| **Processes & Patient Flow:** | | | | | | | |
| - Poor selection of participants for trainings | *“people in the office are the ones who go for TB training, job group P, Q and they are not the ones who are seeing the patients, and they never come and give us the information.”* CO_SSI_31 |  |  |  |  |  |  |
| - Large patient numbers & poor flow & delays | *“sometimes we are overwhelmed by the workload here... as you can see…Chest [clinic]…there is the TB lab guys, sometimes they do [specimen collection] ...if a child is really sick…they are usually done the gastric lavage, from the ward”* CO_SSI_23 |  |  |  |  |  |  |
| - Lack of dedicated spaces for specimen collection | *“Sample collection is usually done at the lab…sometimes the children are sent to the paediatrics ward [from OPD] for collection...”* NO_SSI_19 |  |  |  |  |  |  |
| **Resources:** | | | | | | | |
| - Skilled manpower | *“I’m not comfortable, I request for radiologist’s report which can take you even up to two weeks”* CO_SSI_31 |  |  |  |  |  |  |
| - Equipment & reagents | *“Most times no coz sometimes we have stock outs of Xpert …”* MO_SSI_14 |  |  |  |  |  |  |
| - Guidelines & job aides | *“No, we don’t have charts. In my place we don’t have charts...”* Paed_SSI_09 |  |  |  |  |  |  |
| **WIDER HEALTH SYSTEM (COMMUNITY& POLICY) INFLUENCERS** | | | | | | | |
| **Implementation of TB programme directives:** | | | | | | | |
| - On training | *“We realized that when we rolled out Xpert we focused a lot of our training in the lab personnel, thereby leaving out the drivers of the service use…”* KII_01 |  |  |  |  |  |  |
| - On resources | *“government or the hospital is very supportive. Because for like chest x-rays and such it’s not very expensive. But like we have said for Xpert it’s free. You don’t pay for it...”* Interns SGD |  |  |  |  |  |  |
| - On processes e.g. referrals, decentralisation, case finding, many registers/forms to fill | *And those forms are very annoying to fill [[laughter]] They are very long, they are very long…You get here you are tired…you have a kiform to fill and you are not just filling one…”* COI_SSI_21 |  |  |  |  |  |  |
| - On data use for audit, supervision | *“we do data quality audits and they are done together with the health care workers so it is a participatory sort of quality audit. And the feedback is given on the spot…”* KII_01 |  |  |  |  |  |  |
| **Community Beliefs & Practices:** | | | | | | | |
| - Awareness of TB manifestations in children | *“we encourage them that if you are having a child who is not growing, as a child should be… please bring the baby to the…to be checked…to be screened “KII_02* |  |  |  |  |  |  |
| - Health-seeking behaviour | *“…parent believes that the child has been bewitched… coz she went to the hospital several times, she found there was no improvement...so would give herbs…”* Paed_SSI_03 |  |  |  |  |  |  |
| - Stigma | *“people thinking TB is equal to HIV, so when now someone has to…been told that they have TB now everyone thinks that they are HIV positive…shunned by the family.”* Paed_SSI_03 |  |  |  |  |  |  |
