# Supplementary material for: Perspectives and practices of health workers around diagnosis of paediatric tuberculosis in hospitals in a resource-poor setting – modern diagnostics meet age-old challenges
Source: BMC Health Serv Res. 2020 Aug 1;20:708. doi: 10.1186/s12913-020-05588-6 (PMC7395417; doi:10.1186/s12913-020-05588-6)
Supplement: Supplementary file 4 — Additional file 4. Summary of interviewees. [file 12913_2020_5588_MOESM4_ESM.docx]

## Summary of interviewees

| Code | Gender | Designation/Cadre | County | Interview type |
| --- | --- | --- | --- | --- |
| PHASE 1 | | | | |
| Paed_SSI_001 | M | Paediatrician | H | Semi-structured interview |
| Paed_SSI_002 | F | Paediatrician | B | Semi-structured interview |
| Paed_SSI_003 | F | Paediatrician | K1 | Semi-structured interview |
| Paed_SSI_004 | F | Paediatrician | K2 | Semi-structured interview |
| CO_SSI_005 | F | Clinical officer | V | Semi-structured interview |
| Paed_SSI_006 | F | Paediatrician | N1 | Semi-structured interview |
| Paed_SSI_007 | F | Paediatrician | N2 | Semi-structured interview |
| Paed_SSI_008 | F | Paediatrician | K3 | Semi-structured interview |
| Paed_SSI_009 | M | Paediatrician | E | Semi-structured interview |
| Paed_SSI_010 | M | Paediatrician | M1 | Semi-structured interview |
| Paed_SGD_011 | M  M | Paediatrician  Paediatrician | V  M2 | Small group discussion |
| Paed_SSI_012 | M | Paediatrician | N1 | Semi-structured interview |
| Paed_SSI_013 | F | Paediatrician | T | Semi-structured interview |
| MO_SSI_014 | F | Medical Officer | M3 | Semi-structured interview |
| PHASE 2 | | | | |
| MPH_KII_015 | F | Public Health Officer | N1 | Key informant in-depth interview |
| NO_SSI_016 | F | Nursing Officer | N1 | Semi-structured interview |
| MO_SSI_017 | F | Medical Officer | N1 | Semi-structured interview |
| NO_SSI_18 | F | Nursing Officer | N1 | Semi-structured interview |
| MOI_SSI_019 | M | Medical Officer Intern | N1 | Semi-structured interview |
| NO_SSI_020 | F | Nursing Officer | N1 | Semi-structured interview |
| COI_SSI_021 | F | Clinical Officer Intern | N1 | Semi-structured interview |
| NO_SSI_022 | F | Nursing Officer | N1 | Semi-structured interview |
| CO_SSI_023 | F | Clinical Officer | N1 | Semi-structured interview |
| CO_SSI_024 | F | Clinical Officer | N1 | Semi-structured interview |
| COI_SSI_025 | M | Clinical Officer Intern | N1 | Semi-structured interview |
| MO_SSI_026 | F | Medical Officer | N1 | Semi-structured interview |
| INT_SDG_027 | M | Medical & Clinical officer Interns | N1 | Small group discussion |
| MOI_SSI_028 | F | Medical Officer Intern | N1 | Semi-structured interview |
| MO_SSI_029 | F | Medical Officer | N1 | Semi-structured interview |
| NO_SSI_030 | F | Nursing Officer | K4 | Semi-structured interview |
| CO_SSI_031 | F | Clinical Officer | K4 | Semi-structured interview |
| MO_SSI_032 | F | Medical Officer | K4 | Semi-structured interview |
| CO_SSI_033 | F | Clinical Officer | K4 | Semi-structured interview |
| MPH_KII_034 | F | Public Health Officer | K4 | Key informant in-depth interview |
| MOI_SSI_035 | F | Medical Officer Intern | K4 | Semi-structured interview |
| INT_SGD_036 | F  M | Clinical Officer Intern  Nursing Officer Intern | K4 | Small group discussion |
| MO_SSI_037 | F | Medical Officer | K4 | Semi-structured interview |
