# Supplementary material for: Perspectives and practices of health workers around diagnosis of paediatric tuberculosis in hospitals in a resource-poor setting – modern diagnostics meet age-old challenges
Source: BMC Health Serv Res. 2020 Aug 1;20:708. doi: 10.1186/s12913-020-05588-6 (PMC7395417; doi:10.1186/s12913-020-05588-6)

Legend
DST: Drug Susceptibility testing
IPT: Isoniazid Preventive Therapy
RTI: Respiratory tract infection
S/S: Signs and symptoms

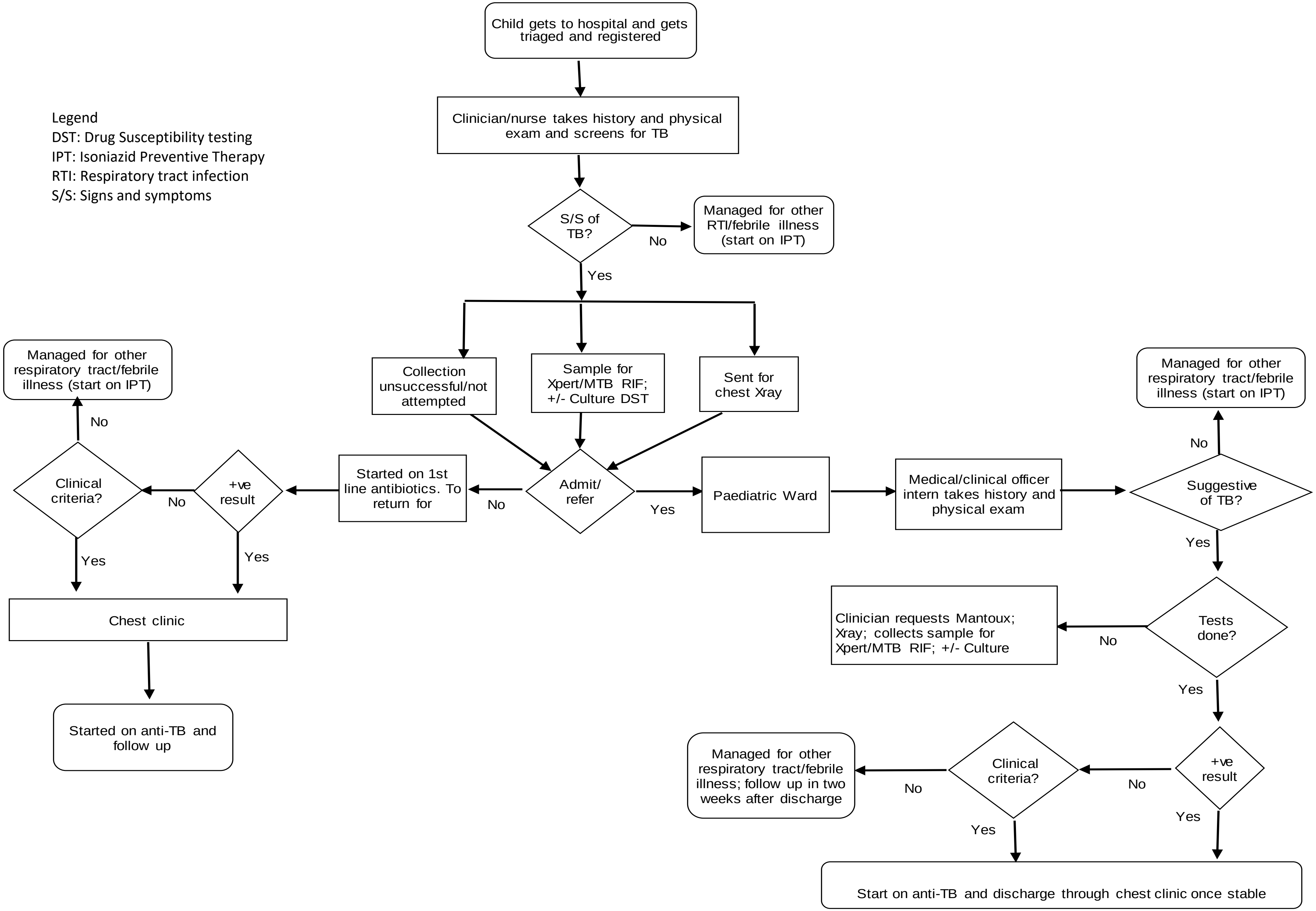

Supplement: Supplementary file 5 — Additional file 5. Patient Flow in a typical Kenyan county hospital. [file 12913_2020_5588_MOESM5_ESM.pdf]
